# Osteopontin promoted cardiac inflammation through increased interleukin-12 in acute myocarditis

**DOI:** 10.1186/s43556-025-00333-z

**Published:** 2025-10-24

**Authors:** Xiang Nie, Jiahui Fan, Yatong Qin, Jianpei Wen, Zhibing Lu, Chen Chen, Dao Wen Wang

**Affiliations:** 1 Division of Cardiology, Department of Internal Medicine and Hubei Key Laboratory of Genetics and Molecular Mechanisms of Cardiological Disorders, Tongji Hospital, Tongji Medical College and State Key Laboratory for Diagnosis and Treatment of Severe Zoonotic Infectious Diseases, Huazhong University of Science and Technology, Wuhan, 430000 China; 2 Department of Cardiology, Hubei Provincial Clinical Research Center for Cardiovascular Intervention, Zhongnan Hospital of Wuhan University, Institute of Myocardial Injury and Repair, Wuhan University, Wuhan, China

**Keywords:** Osteopontin (OPN), Interleukin-12 (IL-12), Myocarditis, Coxsackievirus B3 (CVB3), Signal transducer and activator of transcription 4 (STAT4), Macrophages

## Abstract

Osteopontin (OPN) is a multifunctional glycoprotein known to play critical roles in autoimmunity and tissue repair, yet its function in acute viral myocarditis remains poorly understood. To systematically investigate the pathogenesis of viral myocarditis, this study integrated clinical observations from patients with myocarditis and four genetically distinct mouse strains following intraperitoneal inoculation with coxsackievirus B3 (CVB3). Cardiac transcriptomic profiling via RNA-seq uncovered global changes in gene expression, while quantitative RT-PCR and ELISA assays were employed to measure OPN expression levels in cardiac tissues and plasma. A macrophage-specific OPN knockout (OPN^−/−^) model was generated by crossing OPN flox/flox mice with Lyz2-Cre transgenic mice. In patients with myocarditis, elevated levels of plasma OPN were observed, suggesting its potential utility as a diagnostic biomarker. In murine models, cardiac OPN expression was significantly upregulated during acute viral myocarditis and exhibited a strong inverse correlation with systolic and diastolic function. Further analyses identified macrophages as the primary cellular source of OPN in the heart. Mechanistically, macrophage-derived OPN was shown to enhance the secretion of IL-12, thereby amplifying the local inflammatory response. Genetic ablation of OPN in macrophages markedly attenuated CVB3-induced cardiac dysfunction and reduced myocardial immune cell infiltration. STAT4 was demonstrated to directly bind to the promoter region of OPN and enhance its expression. Correspondingly, pharmacological inhibition of STAT4 using lisofylline significantly suppressed OPN expression in vivo. In summary, OPN functions as a crucial pro-inflammatory regulator in acute viral myocarditis and represents a promising therapeutic target for mitigating virus-induced cardiac damage.

**Supplementary Information:**

The online version contains supplementary material available at 10.1186/s43556-025-00333-z.

## Introduction

Acute myocarditis is an inflammatory condition of the myocardium, marked by the sudden onset of diverse clinical manifestations, including chest pain, dyspnea, and palpitations [1, 2]. Approximately 25% of patients with acute myocarditis develop cardiac dysfunction, ventricular arrhythmias, or acute heart failure [3]. Fulminant myocarditis represents the most severe manifestation of acute myocarditis, distinguished by a rapidly progressing clinical trajectory that can lead to significant hemodynamic instability and potentially result in mortality [4]. Myocarditis progresses through three distinct pathological stages: (1) the acute injury phase, marked by direct virus-induced myocardial damage; (2) the subacute immune phase, predominantly characterized by secondary viral-mediated autoimmune myocardial injury; and (3) the chronic phase, during which a typical presentation of dilated cardiomyopathy emerges as a consequence of extensive myocardial injury [5]. The etiology of myocarditis typically involves infectious agents, such as viruses and bacteria, and non-infectious triggers, including systemic autoimmune disorders and certain medications. Viral infections have been identified as the predominant cause of acute myocarditis [6]. Previous studies have shown that coxsackievirus (especially coxsackie B3 virus, CVB3) is one of the most common pathogenic factors, and usually have been used to induce myocarditis mice models. With the application of clinical myocardial biopsy, it has been found that parvovirus is increasingly prevalent in the occurrence of myocarditis [7]. In recent years, some patients with COVID-19 caused by novel coronavirus (SARS-CoV-2) have developed fulminant myocarditis [8–11]. Since the commencement of the COVID-19 vaccination campaign, there has been a progressive increase in reports of myocarditis associated with the COVID-19 vaccine [12, 13]. Virus-induced cardiac injury primarily involves two mechanisms: [14, 15] First, virus utilizes nucleic acids and proteins within host cells to replicate extensively, leading to host cell rupture, viral release, and subsequent infection of neighboring cells, triggering a cascade reaction. Additionally, proteases encoded by viral genome participate in cleaving host cell proteins, thereby damaging host cells. Second, after viral infection, host's innate and adaptive immune responses are excessively activated, which cause tissue injury. Moreover, in certain patients, inflammation can result in significant scarring, which induces left ventricular remodeling. This process may ultimately lead to dilated cardiomyopathy or, alternatively, to a predominantly hypokinetic nondilated phenotype of cardiomyopathy [16].

Upon infection, immune cells, particularly neutrophils and monocytes/macrophages, are recruited to the affected tissue, where they limit microbial invasion via phagocytosis and antigen presentation. While monocytes/macrophages play a critical role in pathogen containment and clearance, their excessive activation can exacerbate tissue damage during infections and inflammatory conditions [17]. In acute myocarditis, substantial macrophage infiltration into myocardium has been observed, contributing to cardiac injury. Single-cell analyses of cardiac immune cells in autoimmune myocarditis have underscored that macrophage constituted major populations of immune cells and were clustered into 9 clusters identified marker genes for each cluster such as Cxcl9 for cluster 2, Ccl8 for cluster 3, Vcan for cluster 6, and Vsig4 for cluster 8 [18]. Among these, classically activated (M1-like) macrophages promote tissue damage and secrete elevated levels of pro-inflammatory cytokines-such as TNF-α, IL-1α/β, IL-6, IL-12, and IL-23, culminating in a cytokine storm that exacerbates myocardial injury [19, 20].

Osteopontin (OPN), encoded by the secreted phosphoprotein 1 (SPP1) gene, is a multifunctional matricellular glycoprotein secreted by immune cells (e.g., macrophages, T cells) and fibroblasts [21]. OPN exhibits diverse immunomodulatory roles, exerting both pro-inflammatory and anti-inflammatory effects depending on the inflammatory milieu [22]. Previous studies have implicated OPN in promoting pro-inflammatory macrophage polarization, where its upregulation sustains inflammation, while OPN deficiency attenuates inflammatory and fibrotic responses [23, 24]. Conversely, other evidence indicates that OPN enhances pathogen clearance, mitigates tissue damage, and facilitates tissue repair during infections and allergic airway inflammation [25, 26]. In autoimmune myocarditis, the expression of OPN is increased [27], and might sever as a biomarker for inflammatory heart disease [28], but OPN deficient mice are not protected from experimental autoimmune myocarditis [29]. After chronic CVB3 infection, OPN expression was increased and high expression levels of OPN in acute myocarditis are associated with consecutive development of extensive fibrosis and dilated cardiomyopathy [30]. OPN drives right ventricular failure by activating cardiomyocyte inflammatory responses and apoptosis via the integrin-ανβ3/PERK/CHOP pathway, identifying it as a potential therapeutic target [31]. Previous studies provided a spatially resolved atlas of COVID-19 alveolar damage, identifying macrophage-derived SPP1/osteopontin signaling as a key regulator of early pro-inflammatory and pro-fibrotic pathways [32]. While OPN’s involvement in immune regulation and myocarditis were observed, the underlying functions and regulatory mechanisms remains poorly understood.

To better understand the mechanism involved in acute myocarditis, four different animal models that mimic human pathogenesis were constructed on four mouse strains (A/J, C3H, BALB/c and C57BL/6, respectively) induced by CVB3 infection. Our subsequent focus on OPN, one of the conserved genes, was motivated by its central role in inflammatory cascades and newly discovered STAT4-mediated regulatory mechanism. Together, these findings highlight OPN as a potential therapeutic target for mitigating acute myocarditis progression.

## Results

### OPN serves as a biomarker for myocarditis diagnosis

**Fig. 1:**
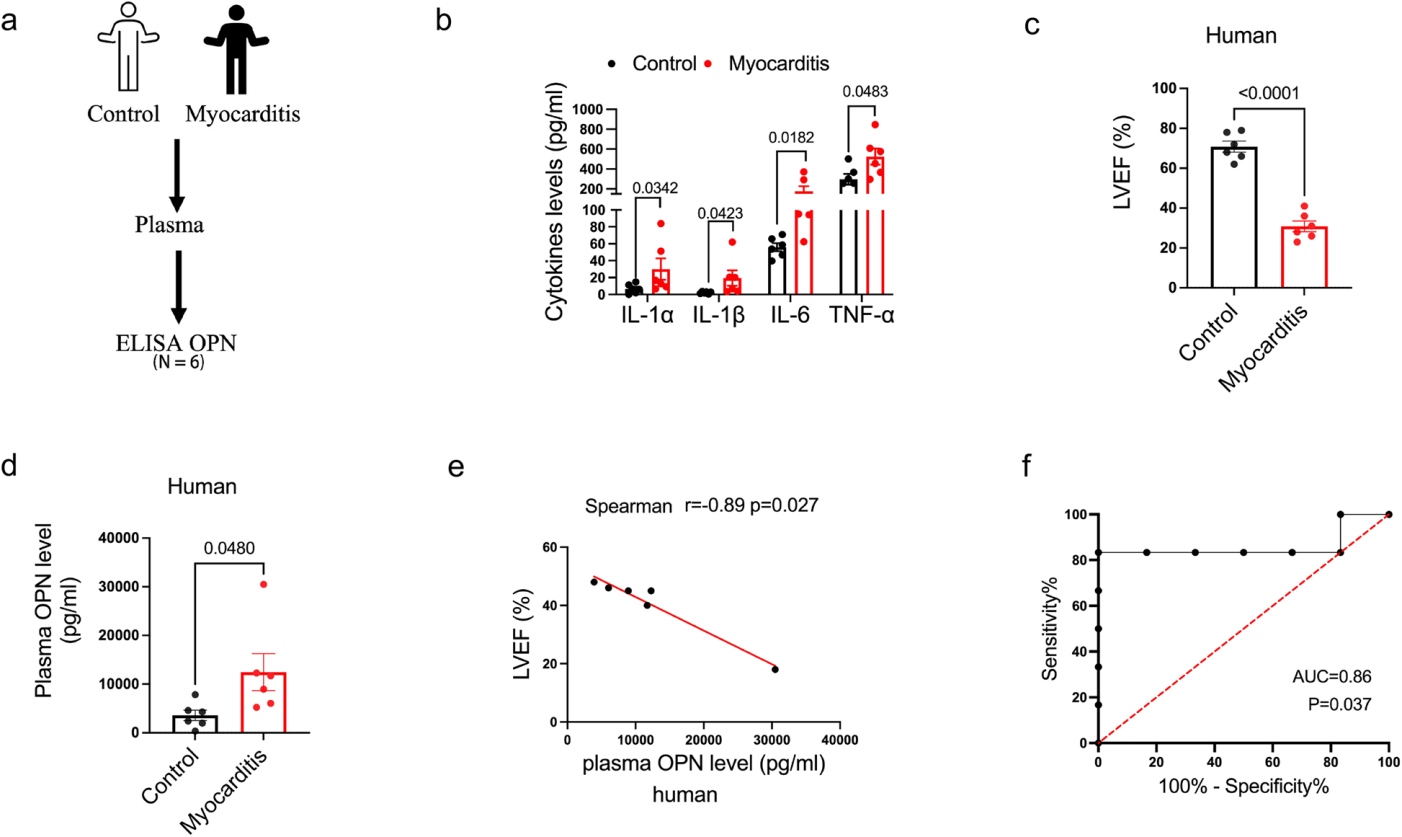
OPN serves as a biomarker for myocarditis diagnosis. **a** Diagram illustrates detection of plasma OPN from acute myocarditis patients. **b** ELISA assay of IL-1-α, IL-1-β, IL-6 and TNF-α expression levels in plasma of acute myocarditis patients. *p*-value analyzed by Unpaired T-Test (*N* = 6). **c** Echocardiographic parameters in acute myocarditis patients and Controls. LVEF: left ventricular ejection fraction. *p*-value analyzed by Unpaired T-Test (*N* = 6). **d** ELISA assay of OPN levels in acute myocarditis patients. *p*-value analyzed by Unpaired T-Test (*N* = 6). **e** Person analyses of correlation between OPN levels and LVEF, respectively (*N* = 6). **f** ROC analyses determine specificity and sensitivity of OPN for acute myocarditis diagnosis (*N* = 6)

In previous study, we employed cytokine array to analyze expression changes of inflammatory factors in the plasma of myocarditis patients [33]. The results revealed significantly elevated levels of OPN in plasma of myocarditis patients. To further evaluate diagnostic potential of OPN in acute myocarditis, we confirmed plasma OPN levels in myocarditis patients and healthy controls (Fig. 1a). The inclusion criteria for patients with myocarditis were according to the Chinese Society of Cardiology expert consensus statement. These patients were clinically diagnosed by sudden onset of disease with inflammation (Fig. 1b) and severe hemodynamic dysfunction (Fig. 1c and Table S1). The plasma of patients with acute myocarditis demonstrated significantly elevated concentrations of OPN in comparison to that of healthy subjects (Fig. 1d and Table S1).

Additionally, a significant inverse association was observed between circulating OPN levels and cardiac function (as assessed by LVEF) in acute myocarditis patients (Fig. 1e and Table S2). No significant correlations were observed between OPN levels and either cTnI or NT-proBNP (Fig. S1a and S1b). Moreover, ROC curve analysis demonstrated excellent diagnostic performance of OPN for acute myocarditis with an AUC of 0.86 (p = 0.037) (Fig. 1f).

These findings indicated that OPN may serve as an independent diagnostic biomarker and play vital roles in the pathogenesis of acute myocarditis.

### OPN is increased in the heart from mice with acute viral myocarditis

**Fig. 2:**
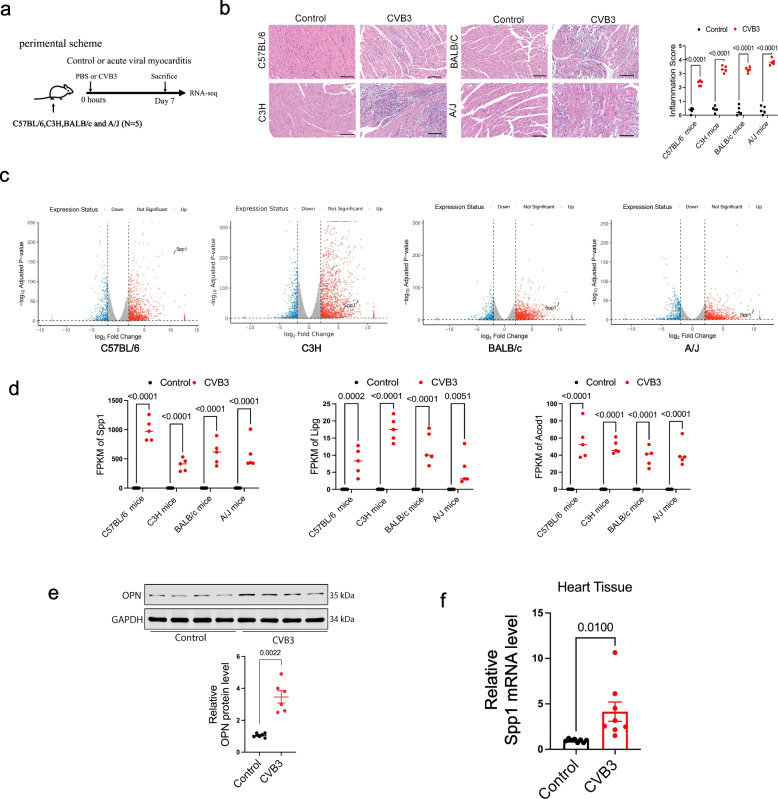
OPN is increased in the heart from mice with acute viral myocarditis. **a** Graphic experimental scheme in acute viral myocarditis mice models. **b** H&E staining of inflammatory cell infiltration in heart tissues after CVB3 infection. *p*-value analyzed by Unpaired T-Test (*N* = 5). **c** Volcano plot for the dysregulated genes detected among four myocarditis mice models. **d** RNA-seq detected FPKM of Spp1, Lipg and Acod1 in heart tissues after CVB3 infection. *p*-value analyzed by Unpaired T-Test (*N* = 5). **e** Western blotting assay of OPN protein level in heart tissues from acute myocarditis (BALB/c mice). *p*-value analyzed by Unpaired T-Test (*N* = 6). **f** The expression of Spp1 was detected by qPCR assay in BALB/c mice after CVB3 infection. The GAPDH was used as housekeeping gene (*N* = 8)

To better understand the mechanism involved in acute myocarditis, four different myocarditis animal models that mimic human pathogenesis were constructed (C57BL/6, C3H, BALB/c and A/J, respectively) by CVB3 infection (Fig. 2a). Severe infiltration of inflammatory cells in the heart tissues of four different mouse strains was observed after CVB3 infection (Fig. 2b). RNA-seq performed in heart tissues showed that more than 2000 genes were differentially expressed in each myocarditis model, and 1440 genes were commonly up-regulated while 787 genes were commonly down-regulated (fold change > 2 and adjusted *p*-value < 0.05) among the four mouse strains after CVB3 infection (Fig. 2c and S1c). RNA-seq analysis identified Spp1, Lipg, and Acod1 among the most significantly and consistently upregulated genes across all myocarditis models, as defined by their high fold-change and statistical significance (Fig. 2d and S1d).

To further prove this, the protein and mRNA levels of OPN in heart after CVB3 infection were determined, finding that OPN expression was significantly increased in acute myocarditis (Fig. 2e and 2f).

These data suggested that OPN might be a vital regulator in acute myocarditis.

### OPN is predominantly expressed by macrophages in acute myocarditis

**Fig. 3:**
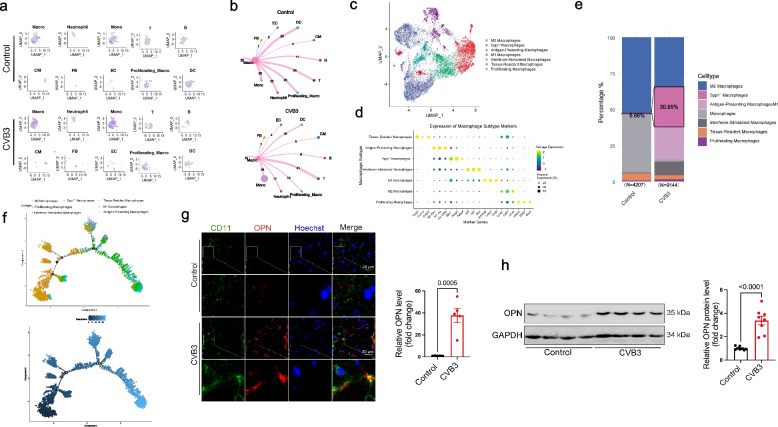
OPN is predominantly expressed by macrophages in acute myocarditis. **a** UMAP visualization showing Spp1 expression in cardiac and immune cells from control and acute myocarditis mice. **b** Interaction number of ligand–receptor pairs between macrophages and other cell populations in the control and myocarditis group. **c** UMAP plots of macrophage subpopulations colored by cell type. **d** Bubble plot of marker gene expression in macrophage subpopulations; dot color indicates the average expression level and size shows the percentage of expressing cells. **e** After CVB3 infection, Spp1 positive macrophages were increased. **f** Developmental trajectories of Macrophage 1–7 colored by pseudotime and Macrophages. **g** IF staining of OPN expression in heart tissues after CVB3 infection. *p*-value analyzed by Unpaired T-Test (*N* = 5). **h** Western blotting assay of OPN protein level in macrophages RAW264.7 after CVB3 infection. *p*-value analyzed by Unpaired T-Test (*N* = 8)

We re-analyzed our previously published single-cell RNA sequencing data [4], which revealed that OPN was predominantly expressed in macrophages and monocytes. We presented UMAP and ridge plots illustrating the normalized expression levels of Spp1 (OPN) across cell types under Control and CVB3 conditions (Fig. 3a and S1e). Furthermore, its expression was significantly upregulated in both macrophages and monocytes after CVB3 infection (Fig. S1f). Monocytes are activated in the circulation, then infiltrate into the heart and differentiate into macrophages to exert their most significant direct effects on cardiac tissue, including phagocytosis, antigen presentation, cytokine-mediated damage, and regulation of fibrosis. Macrophages act as the ultimate functional "executors" of this pathway within the target organ. Thus, we focused on OPN in macrophages in the current study. We further evaluated intercellular communication between macrophages and various cell types by examining interaction weights. Our analysis revealed that macrophages exhibited the most robust interactions with diverse immune cell populations (Fig. 3b). To enhance the understanding of innate immune cell subtypes impacting myocarditis progression, particularly Spp1-expressing macrophages, we performed a re-clustering analysis of macrophages, resulting in the identification of seven distinct clusters (Macrophage 1–7) based on transcriptomic similarities. These clusters were characterized and designated according to their unique gene expression profiles, including a subset of Spp1^+^ macrophages (highest relative expression of Spp1, Fabp5 and Fabp4), Tissue-Resident macrophages (Vsig4, cxcl13, Cd207), antigen-presenting macrophages (H2-Eb1, H2-Aa, H2-DMa), interferon-stimulated macrophages (Ifit3, Ifit2, IFi44), M1 macrophages (S100a8, Ly6c2, Chil3), M2 macrophages (Lyve1, Cd81, Ltc4s) and proliferating macrophages (Ube2c, Mki67, Birc5) (Fig. 3c and 3 d). A comparative analysis of cellular proportions demonstrated a significant expansion of Spp1^+^ macrophages in mice with acute myocarditis (Fig. 3e). Given the substantial proportional changes observed in macrophages during disease progression, we hypothesized that these macrophages exhibit developmental relationships. Trajectory analysis notably revealed that diverse macrophage phenotypes, including Interferon-Stimulated Macrophages, possess the potential to differentiate into Spp1^+^ macrophages (Fig. 3f). To determine the function of macrophages subsets, the differential expression gene was analyzed in Spp1^+^ macrophages as compared to Spp1^−^ macrophages. This subtype of macrophages showed higher expression of Spp1, and genes related to the cytokines as compared to other subpopulations. GO analysis indicated that Spp1^+^ macrophage could regulate inflammatory response and leukocyte migration (Fig. S1g), implying that Spp1^+^ macrophages execute distinct function in acute myocarditis.

To further prove distribution of OPN in macrophages, we isolated primary cardiomyocytes and non-cardiomyocytes (non-CMs) from C57BL/6 mice with or without CVB3 infection (Fig. S1h). Identification of primary cardiomyocytes and non-cardiomyocytes isolated from adult murine hearts were confirmed by qPCR (cTNT for CMs, CD11b and CD68 for Immune cells in non-CMs) (Fig. S1i). qPCR analysis revealed predominant Spp1 expression in non-CMs (Fig. S1j), which was further localized to macrophages by immunofluorescence (IF) (Fig. 3g). Elevated OPN expression was observed in macrophages (RAW264.7) after CVB3 infection, suggesting a potential role for OPN upregulation in macrophages during viral myocarditis (Fig. 3h). These suggested that OPN played vital roles in macrophages.

### OPN regulates migration, apoptosis and IFN signaling pathway in macrophages

**Fig. 4:**
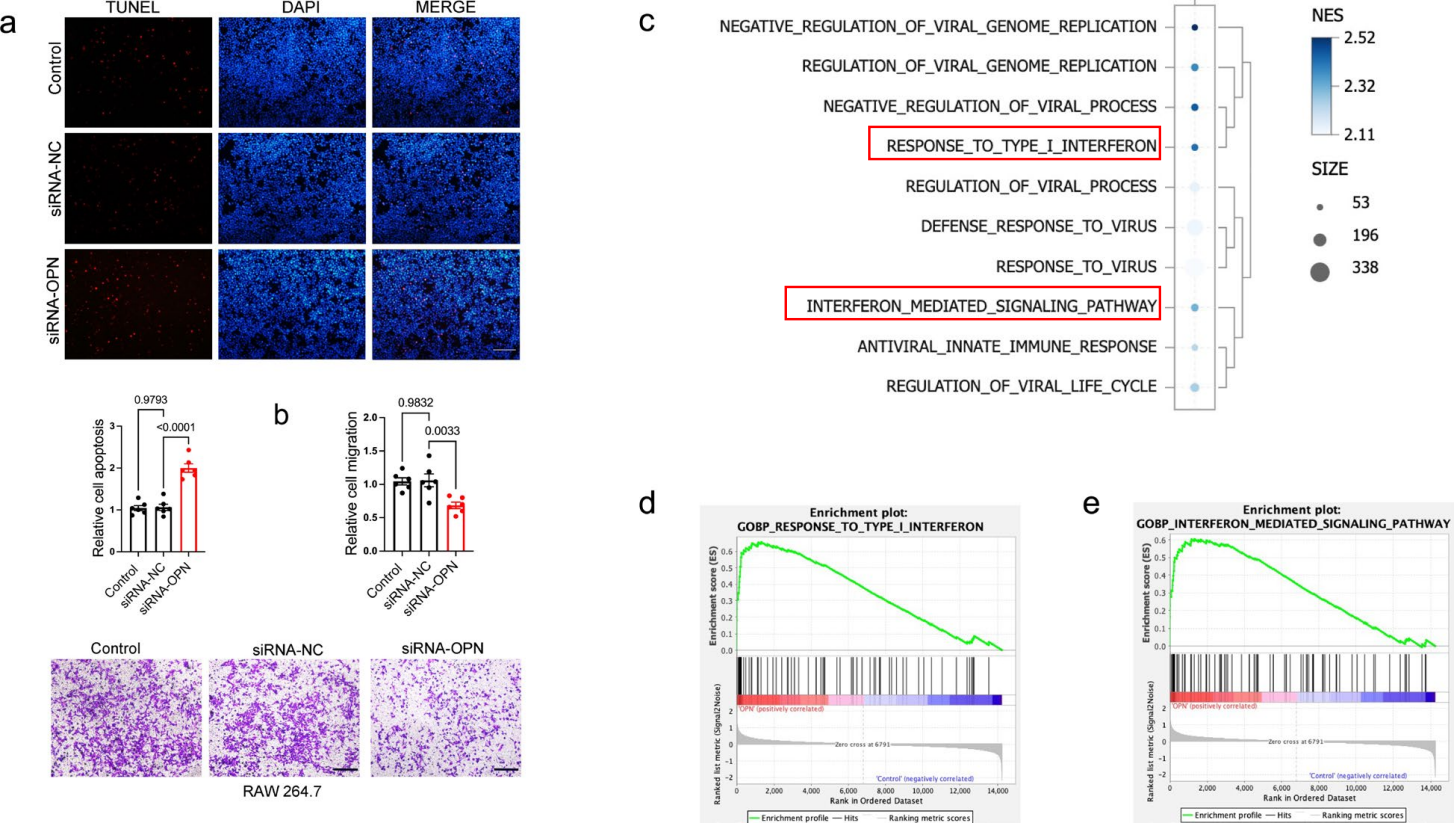
OPN regulates migration, apoptosis and IFN signaling pathway in macrophages. **a** TUNEL staining for apoptosis analysis in AAV-OPN treated macrophages. *p*-value analyzed by Unpaired T-Test (*N* = 6). **b** Cells’ migration detection in AAV-OPN treated macrophages. *p*-value analyzed by Unpaired T-Test (*N* = 6). **c** GSEA analysis for the dysregulated genes in AAV-OPN treated RAW264.7 macrophages. **d** GSEA analysis identified RESPONSE TO TYPE I INTERFERON signal pathway (Normalized Enrichment Score, NES = 2.42; False Discovery Rate, FDR = 0). **e** GSEA analysis identified the NTERFERON MEDIATED SIGNALING PATHWAY (Normalized Enrichment Score, NES = 2.31, False Discovery Rate, FDR = 2.46 × 10^–4^)

To explore the function of OPN in macrophage, endogenic expression of OPN was knockdown in RAW264.7 cells (Fig. S2a). TUNEL assay indicated that OPN knockdown promoted apoptosis of RAW264.7 cells (Fig. 4a). The data showed that silencing OPN inhibited migration of macrophages (Fig. 4b). These results indicated that OPN knockdown inhibited function of macrophage in progress of myocarditis. Previous studies have indicated that OPN expression may facilitate initiation of protective type-1 immune responses following viral and bacterial infections by differentially regulating the production of the cytokine IL-12 [34]. To further support these findings, levels of IL-12 in macrophages was detected after OPN overexpression (Fig. S2b), finding that OPN promoted IL-12 expression in RAW 264.7 (Fig. S2c).

We conducted RNA-seq analysis following OPN overexpression in macrophages (RAW 264.7). Gene Set Enrichment Analysis (GSEA) of Gene Ontology (GO) terms was conducted to identify significantly enriched biological pathways. Based on the results, we concentrated our analysis on the ten pathways exhibiting the highest degree of significant enrichment, as determined by the normalized enrichment score (NES), which correspond to the most biologically pertinent processes within the framework of our experimental context. GSEA data revealed significant enrichment of interferon (IFN)-related pathways and viral infection-associated signaling, highlighting their critical roles in the observed biological response (Fig. 4c and Table S3). GSEA identified a robust and statistically significant upregulation of interferon (IFN)-associated pathways, most notably the RESPONSE TO TYPE I INTERFERON (Normalized Enrichment Score, NES = 2.42; False Discovery Rate, FDR = 0) and the INTERFERON MEDIATED SIGNALING PATHWAY (NES = 2.31, FDR = 2.46 × 10^–4^). These results indicate that genes involved in type I interferon responses are coordinately and highly activated in the experimental condition. The exceptionally high NES values, particularly for the type I interferon response, reflect a strong biological signal, while the FDR values approaching zero underscore the exceptional statistical confidence in this finding. This pronounced enrichment suggests that OPN is likely a key upstream modulator in the pathogenesis of myocarditis, potentially by amplifying the type I interferon signaling axis-a central pathway known to drive inflammatory and antiviral responses that can lead to myocardial injury and dysfunction (Fig. 4d and 4e).

### OPN deficiency alleviates CVB3-induced cardiac inflammation and dysfunction

**Fig. 5:**
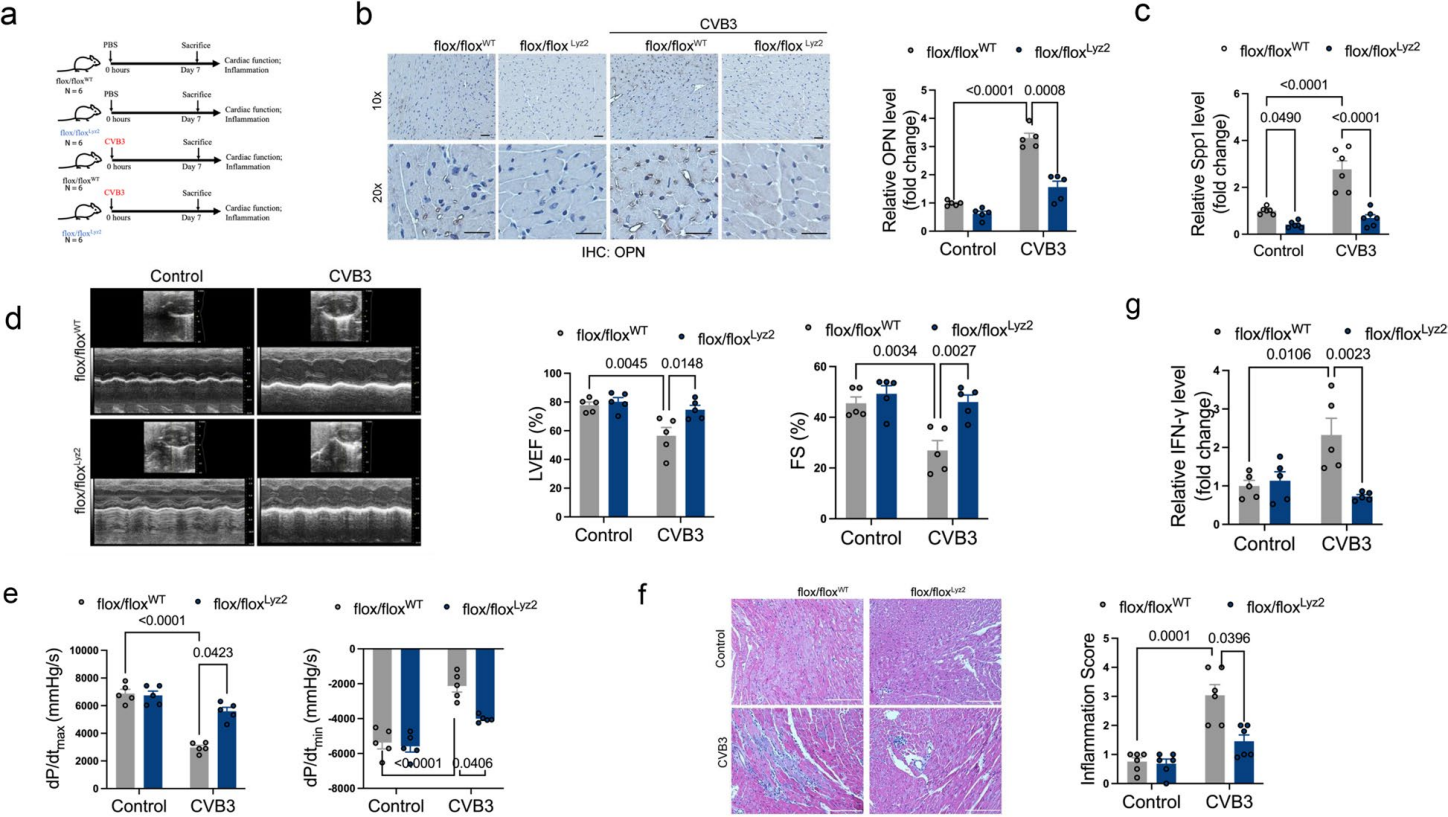
OPN deficiency alleviates CVB3-induced cardiac inflammation and dysfunction. **a** Graphic the experimental scheme of flox/flox^Lyz2^ myocarditis mice. **b** IHC staining of OPN expression in heart tissues after CVB3 infection. *p*-value analyzed by two-way ANOVA with Tukey's multiple comparisons test (*N* = 5). **c** qPCR analysis of OPN expression in flox/flox^Lyz2^ myocarditis mice. *p*-value analyzed by two-way ANOVA with Tukey's multiple comparisons test (*N* = 6). **d** Echocardiographic parameters in flox/flox^Lyz2^ or flox/flox^WT^ mice with Saline or CVB3 infection. LVEF: left ventricular ejection fraction; FS: fractional shorting. *p*-value analyzed by two-way ANOVA with Tukey's multiple comparisons test (*N* = 5). **e** Hemodynamic parameters measured by Millar cardiac catheter system in flox/flox^Lyz2^ or flox/flox^WT^ mice with Saline or CVB3 infection. *dP/dt*_*max*_, peak instantaneous rate of left ventricular pressure increase; *dP/dt*_*min*_, peak instantaneous rate of left ventricular pressure decline. *p*-value analyzed by two-way ANOVA with Tukey's multiple comparisons test (*N* = 5). **f** H&E staining of inflammatory cell infiltration in heart tissue of flox/flox^Lyz2^ or flox/flox^WT^ mice after CVB3 infection. *p*-value analyzed by two-way ANOVA with Tukey's multiple comparisons test (*N* = 6). **g** qPCR analysis of IFN-γ expression in flox/flox^Lyz2^ or flox/flox.^WT^ mice. *p*-value analyzed by two-way ANOVA with Tukey's multiple comparisons test (*N* = 5)

To investigate the functional role of OPN in acute viral myocarditis, we generated macrophage-specific OPN knockout mice by crossing Lyz2-Cre recombinase transgenic mice with floxed OPN (flox/flox) mice, creating flox/flox^Lyz2^ conditional knockout mice (Fig. 5a). Immunohistochemical and qPCR analysis confirmed significant knockout of endogenous OPN expression following CVB3 infection (Fig. 5b, 5c and S3a).

Compared to wild-type controls (flox/flox^WT^), macrophage-specific OPN knockout (flox/flox^Lyz2^) mice exhibited preserved cardiac function after CVB3 infection, as demonstrated by significantly improved left ventricular ejection fraction and fractional shortening (Fig. 5d). In contrast, heart rate remained comparable across all groups, including CVB3-infected fl/fl (non-knockout) controls (Fig. S3b). Hemodynamic assessment further revealed enhanced left ventricular systolic performance in flox/flox^Lyz2^ mice (Fig. 5e). Histopathological analysis showed that OPN deficiency in macrophages attenuated CVB3-induced infiltration of inflammatory cells into myocardial tissue (Fig. 5f and S3c). Furthermore, increased IL-12 mRNA and protein levels induced by CVB3 was significantly attenuated in OPN-deficient mice (Fig. S3d and S3e), but is dispensable for the expression of IL-12 receptors (IL-12RB1 and IL-12RB2) which were unaffected by CVB3 infection (Fig. S3f). A prior study identified IL-12 as a heterodimeric pro-inflammatory cytokine that stimulates the production of interferon-gamma (IFN-γ), a molecule integral to both innate resistance and adaptive immunity [35]. Furthermore, GSEA revealed involvement of IFN-related pathways in OPN-treated macrophages (RAW 264.7). Thus, we detected the expression levels of IFN-γ. Results showed that OPN is required for CVB3-induced IFN-γ production (Fig. 5g) and IL-12 expression. This suggested a potential activation of OPN-IL12-IFN-γ pathway in acute viral myocarditis.

These findings suggest that macrophage-derived OPN contributes to CVB3-induced cardiac dysfunction and inflammatory responses, potentially through upregulation of IL-12 expression. Genetic ablation of OPN in macrophages appears to confer cardioprotective effects during viral myocarditis.

### STAT4 regulates OPN expression in acute myocarditis

**Fig. 6:**
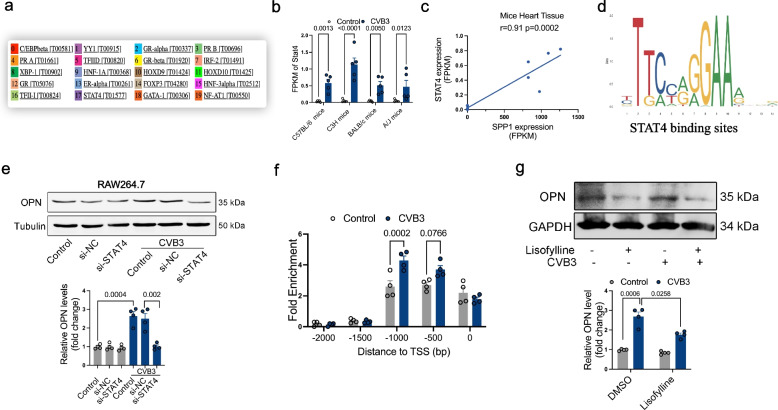
STAT4 regulates OPN expression in acute myocarditis. **a** Transcription factors were predicted using the ALGGE database. **b** RNA-seq detected FPKM of Stat4 in the heart tissues after CVB3 infection. *p*-value analyzed by Unpaired T-Test (*N* = 5). **c** Person analysis of the correlation between Spp1 and Stat4 was analyzed in BALB/c mice with CVB3-induced acute viral myocarditis. **d** The binding sites of STAT4 on Spp1 promoter. **e** Western blotting assay of OPN protein level in macrophage with various treatments. *p*-value analyzed by two-way ANOVA with Tukey's multiple comparisons test (*N* = 4). **f** ChIP-qPCR assay of STAT4 binding on the promoter of Spp1. *p*-value analyzed by two-way ANOVA with Tukey's multiple comparisons test (*N* = 4). **g** Western blotting assay of OPN protein level in macrophage after lisofylline treatment. *p*-value analyzed by two-way ANOVA with Tukey's multiple comparisons test (*N* = 4)

To investigate the transcriptional regulation of OPN, we performed bioinformatic analysis using the ALGGE database (https://alggen.lsi.upc.es), which predicted 20 potential transcription factors with binding sites on the OPN promoter (Fig. 6a). Among these candidates, only STAT4, a known transcriptional activator, exhibited significantly elevated expression in the hearts of acute myocarditis patients, as confirmed by RNA-seq analysis (Fig. 6b and Supplementary Excel). Pearson correlation analysis revealed a positive association between STAT4 and OPN expression (Fig. 6c). Bioinformatic analysis found several binding sites of STAT4 on Spp1 promoter (Fig. 6d and Table S4).

To validate regulatory role of STAT4, we silenced STAT4 in macrophages and observed a marked reduction in OPN levels (Fig. 6e), suggesting that STAT4 positively regulates Spp1 transcription. Chromatin immunoprecipitation followed by qPCR (ChIP-qPCR) further confirmed direct binding of STAT4 to the OPN promoter (Fig. 6f, S3g and S3h). Additionally, pharmacological inhibition of STAT4 using lisofylline significantly suppressed OPN expression in macrophages (Fig. 6g). Collectively, these findings demonstrate that STAT4 drives Spp1 expression in acute myocarditis, highlighting its potential as a therapeutic target for this condition.

## Discussion

In this study, we demonstrated that OPN expression was significantly elevated in acute myocarditis and exhibited strong correlations with both heart impairment and immune activation. Our findings indicate that elevated plasma OPN levels may serve as a complementary diagnostic biomarker for acute myocarditis. OPN deficiency conferred substantial protection against CVB3-induced cardiac inflammation and functional deterioration. Mechanistically, we identified that OPN potentiates interleukin-12 (IL-12) production, and its upregulated expression is mediated through the STAT4 signaling pathway. These results collectively suggested that OPN represents a potential therapeutic target for modulating the progression of acute myocarditis.

OPN is a matricellular protein that mediates cell–matrix interactions and secreted by various cell types, including immune cells. During inflammation, OPN is upregulated in activated macrophages and T cells. Functionally, OPN regulates pro-inflammatory cytokine production and drives macrophage polarization toward the pro-inflammatory M1 phenotype [36]. OPN knockout mice exhibit significantly attenuated lung inflammation and injury compared to wild-type controls [37]. Mechanistically, intracellular OPN enhances interleukin-1β (IL-1β) production via the ERK1/2 and JNK signaling pathways [23]. In the heart, elevated OPN expression is associated with macrophage infiltration, while OPN deficiency improves left ventricular function and reduces cardiac inflammation and fibrosis [30]. In myocarditis, sustained OPN upregulation correlates with progressive fibrosis, highlighting its potential as a therapeutic target to mitigate adverse cardiac remodeling in chronic disease. In this study, we demonstrated that OPN is significantly elevated in acute viral myocarditis and OPN deficiency confers protection against CVB3-induced cardiac inflammation and dysfunction. Furthermore, we found that OPN promotes IL-12 production, though the precise underlying mechanism warrants further investigation.

In addition to its role in inflammation, OPN also plays a crucial role in tissue repair. Plasma OPN levels rise with aging, and OPN deficiency has been shown to attenuate age-related cardiac fibrosis and dysfunction [38]. Following myocardial infarction, cardiac macrophages in the injured heart secrete elevated levels of OPN, and exogenous OPN administration at the ischemic border zone enhances scar formation, mitigates adverse remodeling, and improves overall cardiac function [39]. Conversely, OPN-deficient mice treated with angiotensin II (Ang II) exhibit reduced cardiac fibrosis but impaired systolic function and left ventricular dilatation, underscoring the complex roles of OPN in maintaining cardiac structure and function [40]. Collectively, these findings highlight OPN as a multifunctional cytokine critically involved in cardiac fibrosis and tissue repair. While OPN is a well-established mediator of inflammatory and fibrotic processes in the heart, its expression is not specific to myocarditis. Elevated OPN levels have been consistently documented in patients with acute myocardial infarction, where it is involved in post-infarct remodeling and healing [39]. This presents a significant clinical challenge, as both myocarditis and acute MI can present with similar symptoms, elevated cardiac troponin levels, and even comparable findings on initial imaging. Therefore, the critical diagnostic dilemma often lies not in identifying cardiac injury, but in accurately differentiating between inflammatory and ischemic etiologies. The utility of any proposed biomarker for myocarditis must therefore be evaluated within this specific context of differential diagnosis. Our findings indicate that plasma OPN levels are significantly elevated in patients with acute myocarditis compared to healthy controls, supporting its role in the inflammatory response of the disease. However, its known elevation in myocardial infarction necessitates further research to validate its specificity and utility as a diagnostic biomarker in the crucial differential diagnosis between inflammatory and ischemic cardiac injury.

Viral myocarditis is a well-recognized precursor to DCM and heart failure. However, underline mechanism involved in myocarditis to cardiomyopathy transition is not fully understand. Elevated OPN expression in acute myocarditis is associated with progressive fibrosis, primarily driven by infiltrating macrophages. OPN induces chronic myocarditis leading to dilated cardiomyopathy [41–43]. In OPN-deficient mice, key mediators of extracellular matrix remodeling-including matrix metalloproteinase-3 (MMP-3), tissue inhibitor of metalloproteinases-1 (TIMP1), urokinase-type plasminogen activator (uPA), and transforming growth factor-β1 (TGF-β1) are significantly downregulated, further implicating OPN in cardiac remodeling [44]. In this study, we observed persistently high OPN expression not only during acute myocarditis but also in later stages (30 days post-CVB3 infection) and in DCM. These findings suggested that OPN is instrumental in fibrosis progression and may facilitate the transition from acute myocarditis to DCM. Further investigation is warranted to elucidate the underlying mechanisms.

OPN expression is modulated by diverse stimuli including pressure overload, hypoxia, and immune activation through distinct signaling pathways. Previous studies have demonstrated that vitamin D, interferons, and glucocorticoids can upregulate OPN expression, while protein kinase C suppresses OPN in fibroblasts via epidermal growth factor targeting [45–47]. Additionally, pro-inflammatory cytokines such as tumor necrosis factor-α (TNF-α) and interleukin-6 (IL-6) have been shown to potently stimulate OPN transcription [48, 49]. Signal transducer and activator of transcription 4 (STAT4) plays pivotal roles in immune regulation by activating genes involved in both cell-mediated and humoral immunity. Aberrant STAT4 expression has been documented in autoimmune myocarditis, and the IL-12-STAT4 signaling axis has been implicated in disease pathogenesis. STAT4-deficient mice exhibit resistance to myocarditis [50, 51]. However, the precise role of STAT4-mediated signaling in acute myocarditis remains incompletely understood. STAT4 is a transcription factor activated primarily by interleukin-12 (IL-12), IL-23 and type I interferons (IFNs) [52]. IFN-γ plays a complex and context-dependent role in myocarditis, exhibiting both protective antiviral effects and pro-inflammatory tissue-damaging properties, depending on the disease stage, immune microenvironment, and regulatory factors. In the acute phase of viral myocarditis, IFN-γ is crucial for viral clearance. However, persistent IFN-γ signaling can drive immunopathology. Exacerbates inflammation by recruiting pro-inflammatory macrophages and CD8^+^T cells, leading to myocardial fibrosis and dysfunction [53, 54]. Trajectory analysis notably revealed that Interferon-Stimulated Macrophages may differentiate into OPN^+^ macrophages. Collectively, our data implicate the OPN-IFN-γ signaling axis in the myocarditis.

Upon phosphorylation, STAT4 dimerizes and translocate to the nucleus, where it regulates the expression of genes involved in Th1 cell differentiation, interferon-gamma production, and pro-inflammatory responses. Regarding Lipase G, further investigation may be warranted.

Emerging evidence suggests that STAT4 and OPN interact in multiple biological processes, including T-cell differentiation, cytokine production, and autoimmune disease progression. Both STAT4 and OPN are key players in Th1-mediated immune responses. STAT4 drives Th1 differentiation by upregulating IFN-γ expression, while OPN enhances Th1 responses by sustaining IFN-γ production and inhibiting Th2 cytokines like IL-10 [55, 56]. Studies suggest that OPN may amplify STAT4 signaling by promoting IL-12 secretion from dendritic cells (DCs) and macrophages, further reinforcing Th1 polarization. Additionally, OPN has been shown to interact with STAT4 in the context of Th17 responses. In autoimmune diseases such as multiple sclerosis (MS) and rheumatoid arthritis (RA), both STAT4 and OPN contribute to Th17 cell expansion and IL-17 production, exacerbating inflammation [57]. OPN can modulate STAT4 activation indirectly by influencing upstream cytokines like IL-12 and IL-23, creating a feedback loop that sustains chronic inflammation. In our study, we found that STAT4 upregulates OPN in macrophages. OPN enhances IL-12 production, which in turn activates STAT4, creating a pro-inflammatory loop.

Given their roles in autoimmunity, targeting STAT4 and OPN might be beneficial in autoimmune diseases. STAT4 inhibition reduced IFN-γ and Th1 responses, while OPN-neutralizing antibodies or siRNA-based approaches showed promise in preclinical models of MS and RA [58, 59]. STAT4 might be a potential therapeutic target in immune-related diseases. However, due to the extensive regulatory roles of transcription factors, targeting STAT4 could result in unintended side effects. Notably, STAT4 has been implicated in the exacerbation of chronic CVB3 myocarditis, indicating that therapeutic strategies aimed at STAT4 might inadvertently aggravate prevalent viral infections such as CVB3, thereby exacerbating chronic inflammatory cardiac conditions [51]. Thus, it is imperative to conduct an in-depth examination of the pathophysiological mechanisms underlying acute myocarditis to identify effective therapeutic targets.

In the present study, we observed significant upregulation of STAT4 in CVB3-induced acute myocarditis. Treatment with lisofylline, an of inhibitor STAT4, effectively attenuated inflammation and downregulated OPN expression. Importantly, we identified STAT4 as a direct transcriptional regulator of OPN in macrophages during acute myocarditis, providing novel mechanistic insights into disease progression.

Based on the previous information and our findings, in the current study, we further explored the sources, causes, and specific mechanisms of elevated OPN. The novel contributions of our research are: 1) utilization of multiple mouse strains to identify a key regulator of inflammation across various myocarditis subtypes; 2) the elevated expression of OPN was observed in the early stage of acute viral myocarditis mice and patients with myocarditis; 3) the elevation of OPN in myocarditis was primarily originated from macrophages; 4) conditional knockout of OPN in macrophages prevented virus-induced cardiac injury; 5) STAT4-induced OPN increased IL-12 production, which in turn activated STAT4, generating a pro-inflammatory loop.

One limitation of our study is that, although we primarily focused on the STAT4-OPN axis, we included preliminary data showing a significant increase in Lipase G expression in acute myocarditis, which warrants further validation. Future studies should specifically address: (1) the molecular mechanisms underlying OPN-mediated regulation of IFN-γ, and (2) the therapeutic potential of targeting this pathway in experimental models of myocarditis.

## Materials and method

### Study design

This study was conducted at the Hubei Key Laboratory of Genetics and Molecular Mechanisms of Cardiological Disorders, Huazhong University of Science and Technology. All experiments were performed and analyzed with operators blinded to the genotype, treatment and group information. The animal randomization was performed using a random number generator (http://www.randomizer.org). Animals with similar date of birth or body weight were randomly assigned to each experimental group.

### Human plasma samples

The inclusion criteria for patients with myocarditis were according to the 2013 European Society of Cardiology position statement, 2017 Chinese Society of Cardiology expert consensus statement, and 2009 International Consensus Group on Cardiovascular Magnetic Resonance in Myocarditis statement. These patients were clinically diagnosed by sudden onset of disease, obvious initial symptoms of viral infection, rapid emerging severe hemodynamic dysfunction, inflammation, and diffuse ventricular wall motion decrease as well as exclusion of myocardial infarction. This study was approved by the Ethics Review Board of Tongji Hospital and Tongji Medical College (ID: TJ-C20160202) and conformed to the principles of the Declaration of Helsinki. Informed consents were signed by the subjects recruited in the study or by family members in case of incapacity. The plasma samples were collected from twelve myocarditis patients and six healthy controls. The detail information of patients with acute myocarditis was listed in Table S1 and Table S2.

### ELISA assay

Plasma or cellular levels of OPN and cytokines, including IL-1β (Cat# EK0389), IL-1α (Cat# EK0392), IL-6 (Cat#EK0389), and TNF-α (Cat#EK0525), were measured using the OPN ELISA kit or corresponding cytokine ELISA kits (Boster, China) following the instruction. Briefly, 100 μL standard samples or plasma were added into each microtiter plate well in triplicate and incubated for 90 min at 37 °C. The liquid in each well was removed and 100 μL biotin-labeled antibody dilution was added and incubated for 60 min at 37 °C. Next, 100 μL avidin-peroxidase complex (ABC) dilution was added into each well for 30 min, the wells were washed and colored by incubation with tetramethyl benzidine (TMB) solution at 37 °C. Then, the reactions were stopped, and the absorbance values were immediately measured by a microplate reader. The standard curve was plotted using the concentration of the standard samples, OPN or cytokines concentration in plasma was calculated according to the formula of the standard curve as described previously [60].

### Immunofluorescent staining

Immunofluorescence for OPN and CD11b (both 1:200) was performed on 4-μm cardiac sections using standard protocols, with Alexa Fluor 594-conjugated secondary antibody (1:500). OPN antibody specificity was validated by controls in OPN-modulated macrophages and with primary antibody omission (Fig. S4a and S4b).

### Isolation of CMs and non-CMs from heart tissues

To explore the expression of OPN in cardiomyocytes, the primary cardiomyocytes were isolated from CVB3-infected or control mice, respectively. Briefly, the heart was removed and immersed into ice-cold Tyrode’s buffer after mouse was anesthetized with a mixture of pentobarbital and heparin. The heart was perfused with Tyrode buffer for 5 min at 37 °C, then switched to enzymatic buffer for 20 min. The ventricle part was cut into small tissue pieces and was pipetted gently. Cardiomyocytes were collected by centrifuging at 500 g for 1 min as described previously [61].

### RNA sequencing analysis

To evaluate the mechanism involved in the progress of myocarditis, heart tissues after CVB3 infection were collected from BALB/c, A/J, C3H and C57BL/6 mice, respectively (*N* = 5 in each group) or RAW264.7 cells treated by AAV-OPN (*N* = 4) or AAV-GFP (*N* = 4). RNA isolation, quality control, library construction, and sequencing were performed by Personal Biotechnology Co. (Shanghai, China). Sequencing libraries were performed on a Hiseq X ten platform/NovaSeq 6000 (Illumina, San Diego, CA, USA). The *P*-values were adjusted using Benjamini and Hochberg’s approach to control for the false discovery rate. Genes with an adjusted P < 0.05 and absolute LogFC > 1 measured by DESeq2 were assigned as differentially expressed [62].

### RNA extraction and real-time PCR

Total RNA was isolated from heart tissues or treated cells using TRIzol reagent (Invitrogen, Cat#15,596,026). Following the manufacturer's instructions, RNA was reverse transcribed into cDNA using Hifair III 1 st Strand cDNA Synthesis Super Mix (Yeasen, Cat#11120ES60). Quantitative real-time PCR was performed in triplicate with HieffUNICON® Universal Blue qPCR SYBR Green Master Mix (Yeasen, Cat#11184ES08) on a 7900HT FAST system (Life Technologies). The PCR protocol included 40 cycles of 95 °C for 15 s and 60 °C for 1 min. Relative gene expression was calculated using the 2–ΔΔCt method, as previously described [63], with the corresponding primer sequences listed in Table S5.

### Western blotting assay

To assess OPN expression, protein was extracted from cultured cells and frozen tissues using ice-cold lysis buffer. After quantifying concentrations with a BCA assay kit (Beyotime, Cat# P0009), equal amounts of protein were separated by 10% SDS-PAGE and transferred to PVDF membranes. The membranes were blocked with 5% BSA, followed by incubation with primary antibodies against OPN and GAPDH at 4 °C overnight. Subsequently, a peroxidase-conjugated secondary antibody (ABclonal, Cat# AS014) was applied for 2 h at room temperature. Protein bands were visualized by enhanced chemiluminescence and quantified using ImageJ software (National Institutes of Health). The expression level of OPN was normalized to that of the internal control GAPDH for each sample, as previously described [63, 64].

### Single cell sequencing

Single cells capture and library construction of cardiac immune cells were performed using Chromium Next GEM Single Cell Reagent Kits (SeekGene, Beijing, China). Sequencing was performed on a NovaSeq platform (Illumina, San Diego, USA). Single-cell transcriptome data of immune cells were analyzed using the Cell Ranger Software Suite to perform alignment, filtering, barcode separation, and unique molecular identifier (UMI) counting using default parameters as described previously [4]. Single-cell RNA sequencing was performed on cardiac cells isolated from heart tissues from 6-week-old male A/J mice (*N* = 2 in each group). Cell–cell communication networks were inferred using the R package CellChat (v2) with the mouse ligand-receptor database (CellChatDB.mouse). Key interactions were visualized using the netVisual_circle function. To infer the developmental trajectory of macrophages, we performed pseudotime analysis using the Monocle 2 R package (v2.34.0). A CellDataSet object was created from the raw count data of the selected cell populations. Genes used for ordering the cells along the trajectory were selected based on high variance across the data. The dimensionality was reduced using the DDRTree algorithm, and cells were subsequently ordered along the inferred trajectory to determine their pseudotime values.

### Generation of OPN knockout mice

To explore the function of OPN in the heart, macrophage specific OPN knockout mice was generated in C57BL/6 J mice (Cyagen, Suzhou, China). flox/flox mice were generated using the clustered regularly interspaced palindromic repeat (CRISPR)/CRISPR-associated protein 9 (Cas9) system. flox/flox was cross breeding with Cre (Lyz2) mice to get flox/flox^Lyz2^ mice. The mice were housed at the animal care facility of Tongji Medical College at 25 °C with 12/12 h light/dark cycles and allowed free access to normal mice chow and water throughout the study period. At six-week-old, the flox/flox^Lyz2^ mice was infected by the CVB3, seven days later, cardiac function was analyzed by the echocardiography.

### Echocardiography analysis

Cardiac function was analyzed by echocardiography using the VisualSonics Vevo 1100 (VisualSonics, Toronto, ON, Canada). Myocarditis mice were sedated with 3% isoflurane (Baxter International, Deerfield, Illinois, USA) and fixed in dorsal position on a heated pad (VisualSonics, Toronto, Ontario, Canada). M-mode images of the maximum dimension of the left ventricular (LV) in parasternal short or long axis view were acquired. All acquired images were digitally stored in raw format (DICOM) for further offline analyses. Image analyses were performed using the Vevo LAB Version 3.0 software. Cardiac parameters such as ejection fraction (EF) and fractional shortening (FS) were evaluated in acquired M-mode images as described previously [66].

### Invasive left ventricular hemodynamic measurements

Cardiac function was further measured by Millar catheter. Briefly, Myocarditis or Control mice were anesthetized with a combination of ketamine (100 mg/kg) and xylazine (5 mg/kg) via intraperitoneal injection. A 1.4F Millar catheter (Millar Instruments, Inc., Houston, TX) was inserted into the right carotid artery and advanced into the left ventricular (LV) cavity. LV systolic pressure (LVSP) and LV end-diastolic pressure (LVEDP), maximum and minimum rates of change in LV pressure (dP/dt_max_ and dP/dt_min_, respectively) were recorded as described previously [63].

### Chromatin immunoprecipitation (ChIP)-PCR

ChIP assay was performed using the ChIP assay kit (Beyotime, Shanghai, China; Cat#P2078). Briefly, chromatin was prepared from RAW264.7 cells following crosslinking with formaldehyde, then the DNA was sheared to almost 500 bp size using the micrococcal nuclease (NEBs). The chromatin was immunoprecipitated with anti-STAT4 overnight at 4 °C. Next day, the chromatin was incubated with Protein G Dynabeads for 2 h. After washed with low salt, high salt, and TE buffer, the chromatin was treated with Proteinase K and reverse crosslinked for 6 h at 65 °C. Qiaquick PCR purification kit (Qiagen; Cat#28,104) was used to purify the DNA. Target amplification and detection were performed on a 7900HT FAST real-time PCR system (Life Technologies, Carlsbad, CA). Results were determined by using the 2^−ΔΔCt^method relative to the input DNA as described previously [66]. The primers for qPCR detection were listed in Table S6.

### Statistical analysis

Data are shown as means ± SEM, unless otherwise stated. Statistical tests were performed using GraphPad Prism (v8.0) (GraphPad Software, San Diego, CA). A p value less than 0.05 is considered statistically significant. Shapiro–Wilk test was used for normality analysis. Statistical analyses were then performed with Two tailed student’s t-test (parametric unpaired or paired, two group of analysis), one-way ANOVA with Dunnet’s multiple comparisons test (comparisons between more than two groups, non-parametric unpaired). For data that failed normality testing, Mann–Whitney U test (2 groups), or Kruskal–Wallis with Dunn post-test (3 or more groups) was performed. Two-way ANOVA followed by Dunnett post hoc test were used in the in vivo studies on the same continuous mice when multiple time points were involved. To assess the significance of the correlations, spearman rank correlation coefficient was calculated. The ROC analysis and Area under the curve (AUC) was performed using the GraphPad Prism 8.0 (GraphPad software, CA). A *p*-value less than 0.05 was considered statistically significant. The optimal cutoff for OPN was 7853 pg/mL.

## Supplementary Information

Supplementary Material 1.

Supplementary Material 2.

## Data Availability

The data that support the findings of this study are available on request from the corresponding author upon reasonable request.
