# Supplementary material for: Osteopontin promoted cardiac inflammation through increased interleukin-12 in acute myocarditis
Source: Mol Biomed. 2025 Oct 24;6:85. doi: 10.1186/s43556-025-00333-z (PMC12612485; doi:10.1186/s43556-025-00333-z)
Supplement: Supplementary file 1 — Supplementary Material 1. [file 43556_2025_333_MOESM1_ESM.docx]

**Osteopontin promoted cardiac inflammation through increased interleukin-12 in acute myocarditis**

Xiang Nie^1,2^*, Jiahui Fan^1^*, Yatong Qin^1^, Jianpei Wen^1^, Zhibing Lu^2^, Chen Chen^1#^, Dao Wen Wang^1#^

^1^Division of Cardiology, Department of Internal Medicine and Hubei Key Laboratory of Genetics and Molecular Mechanisms of Cardiological Disorders, Tongji Hospital, Tongji Medical College and State Key Laboratory for Diagnosis and Treatment of Severe Zoonotic Infectious Diseases, Huazhong University of Science and Technology, Wuhan 430000, China.

^2^Department of Cardiology, Hubei Provincial Clinical Research Center for Cardiovascular Intervention, Zhongnan Hospital of Wuhan University, Institute of Myocardial Injury and Repair, Wuhan University, Wuhan, China.

^#^Corresponding author:

Dao Wen Wang (email: [dwwang@tjh.tjmu.edu.cn](mailto:dwwang@tjh.tjmu.edu.cn)). Division of Cardiology, Department of Internal Medicine, Tongji Hospital, Tongji Medical College, Huazhong University of Science & Technology, 1095# Jiefang Ave., Wuhan 430030, China. Tel/fax: 86-27-8366-3399

Chen Chen (email: [chenchen@tjh.tjmu.edu.cn](mailto:chenchen@tjh.tjmu.edu.cn)). Division of Cardiology, Department of Internal Medicine, Tongji Hospital, Tongji Medical College, Huazhong University of Science & Technology, 1095# Jiefang Ave., Wuhan 430030, China. Tel/fax: 86-27-6937-8422

*X.N. and J.F. contributed equally to this article.

**Supplementary methods**

**Materials**

Fetal bovine serum (FBS) and Dulbecco’s Modified Eagle’s Medium (DMEM) were obtained from Transgene Biotech (Beijing, China; Cat# FI101-01). Antibodies against OPN (Cat# A19092) and GAPDH (Cat# AC002) were purchased from ABclonal (Wuhan, China). AAV-GFP or AAV-OPN plasmid were designed and constructed by HANBIO (Shanghai, China).

**Cells cultures and treatments**

RAW264.7 cells were cultured in Dulbecco's Modified Eagle Medium (DMEM), supplemented with 10% fetal bovine serum (FBS) and 1% penicillin-streptomycin. Cells were maintained at 37 °C in a humidified atmosphere containing 5% CO_2_. The medium was refreshed every 2-3 days, and cells were passaged at 80-90% confluence. Cells were transfected with Spp1-specific siRNA or control siRNA using Lipo 2000 Transfection Reagent, strictly according to the manufacturer's instructions. The siRNA-lipid complex was prepared at a final siRNA concentration of 50 nM and added to the cells. The transfection mixture was replaced with complete growth medium 6 hours after transfection. Knockdown efficiency was validated by qRT-PCR analysis 48 hours after transfection.

**Generation of myocarditis mice**

Animal experiments were conducted obeying the guidelines with the approval of the Institutional Animal Care and Use Ethics Committee of the Tongji Medical College. All animal experimental protocols in this study complied with the Guide for the Care and Use of Laboratory Animals published by the National Institutes of Health and were approved by the Institutional Animal Research Committee of Tongji Medical College. Six-week-old male C57BL/6, BALB/c, C3H mice were purchased from Beijing Vital River Laboratory Animal Technology (Beijing, China). A/J mice was obtained from GemPharmatech (Nanjing, China). All four mice strains were randomly assigned to two groups (CVB3 vs. Control). A/J mice and BALB/c mice were intraperitoneally injected with 10^4^ PFU CVB3, while C57BL/6 and C3H mice were intraperitoneally injected with 10^5^ PFU CVB3 as described previously,^61-63^ while phosphate-buffered saline (PBS) was used as the negative control. Day 7 after virus injection*,* all animals were anesthetized with intraperitoneal injections of a mixture of xylazine (5 mg/kg) and ketamine (80 mg/kg). Echocardiography was performed and the mice were sacrificed. The organs were embedded in paraffin or frozen in liquid nitrogen, followed by storage at -80 °C.

**Inflammatory scores analysis**

The inflammatory scores were defined as reported previously^61^ as: 0 score, no inflammatory infiltrates; 1 score, small foci of inflammatory cells between myocytes; 2 score, larger foci of inflammatory cells; 3 score, > 10% of a cross-section involved; 4 score, > 30% of a cross-section involved. Six views of one H&E-stained section under a microscope were randomly collected and averaged as one data point. H&E staining and myocarditis scoring were performed in five mice in each group.

**TUNEL assay**

The TUNEL (Terminal deoxynucleotidyl transferase dUTP Nick End Labeling) assay was performed to detect DNA fragmentation characteristic of apoptotic cells in RAW264.7 cells using the TUNEL kit from Boster Biological Technology, (Cat#MK1012-100, Wuhan, China) according to the manufacturer's instructions, with the following detailed procedure: RAW264.7 cells were cultured in DMEM and seeded at a density of 1 x 10^5^ cells/well in a 24-well plate containing sterile glass coverslips and allowed to adhere overnight. Cells were then treated with siRNA-OPN or siRNA-NC. After treatment, cells were washed gently once with 1 X PBS, fixed by incubating with 4% formaldehyde in PBS for 10 min at room temperature. Fixed cells were permeabilized by incubating with 0.3% Triton X-100 in PBS for 15 min on ice. For positive control: one set of slides was treated with Recombinant DNase I for 15 min at room temperature, then was washed with PBS. For negative control, the terminal deoxynucleotidyl transferaseenzyme will be omitted in a later step. The TUNEL reaction mixture was prepared on ice, involving mixing the TdT enzyme with the reaction buffer containing fluorescently-labeled dUTP, then was added to each coverslip. The plate was incubated in a dark, humidified chamber at 37 °C for 60 min. For the negative control, the TUNEL reaction mixture was prepared without the TdT enzyme and applied to a separate sample. After incubation, the reaction mixture was aspirated, and cells were rinsed three times with PBS to stop the reaction. To visualize all nuclei, cells were incubated with DAPI in PBS for 5 min at room temperature in the dark. Cells were washed twice with PBS. Cells were visualized using a microscope. TUNEL-positive nuclei were identified by red fluorescence, while all nuclei were identified by blue fluorescence. For each condition, five random fields of view were captured. The number of TUNEL-positive nuclei and the total number of DAPI-positive nuclei were counted using image analysis software. The results are expressed as the mean percentage from six independent experiments.

**Migration assay**

The migratory capacity of RAW264.7 cells treated by siRNA-OPN or siRNA-NC was assessed using a transwell migration assay. The procedure was performed as follows: Cells were starved in serum-free DMEM to minimize the effect of proliferation. After starvation, cells were harvested using 0.25% Trypsin-EDTA, washed twice with PBS, and resuspended in the starvation medium. The lower chamber of the 24-well plate was filled with 400 µL of DMEM with 10% FBS. The prepared transwell insert was carefully placed into the well. 100 µL of the treated cell suspension was gently added to the upper chamber of the insert. The assembled plate was incubated under standard cell culture conditions for 12-24 hours to allow cell migration. After incubation, the transwell inserts were carefully removed from the plate. Using a cotton swab, the non-migrated cells remaining on the upper surface of the membrane were gently but thoroughly wiped away. The inserts were then rinsed briefly in 1X PBS to remove any loose cells or debris. Cells that had migrated to the lower surface of the membrane were fixed by immersing the insert in 4% formaldehyde in PBS for 15 min at room temperature. After fixation, the inserts were washed gently with PBS. Migrated cells were stained with 0.1% crystal violet solution for 20 min at room temperature. After staining, the inserts were washed thoroughly with distilled water to remove excess dye and allowed to air dry completely. The dried inserts were visualized under a microscope. The number of migrated cells was quantified by counting the stained cells in five random fields of view per insert. For each experimental condition, the assay was repeated in six independent experiments. Data are presented as the mean number of migrated cells per field of view ± SEM.

**Hematoxylin and eosin (H&E) staining**

Heart tissues from the four mice strains were fixed with 4% paraformaldehyde, embedded in paraffin, and sectioned into 4 μm slices. Cardiac morphology was evaluated by hematoxylin and eosin (H&E) staining and measured using Image-Pro Plus Version 6.0 software (Media Cybernetics, Washington, USA). Six views of one H&E-stained section under a microscope were randomly collected and averaged as one data point. For each group, the results were analyzed using at least six groups of data.

**Gene Set Enrichment Analysis**

GSEA of Gene Ontology (GO) terms was conducted to identify significantly enriched biological pathways. Using the GSEA software (v4.4.0) with the c5.go.v2023.1.Hs.symbols.gmt gene set database, we analyzed gene expression profiles ranked by signal-to-noise ratio. The analysis employed 1,000 phenotype permutations for statistical validation, with significance thresholds set at FDR < 0.25 and nominal p-value < 0.05. From the results, we focused on the top 10 most significantly enriched pathways based on normalized enrichment score (NES), which represent the most biologically relevant processes in our experimental context. Leading edge analysis further identified key driver genes within these top pathways.

**Supplementary Figure 1**

**
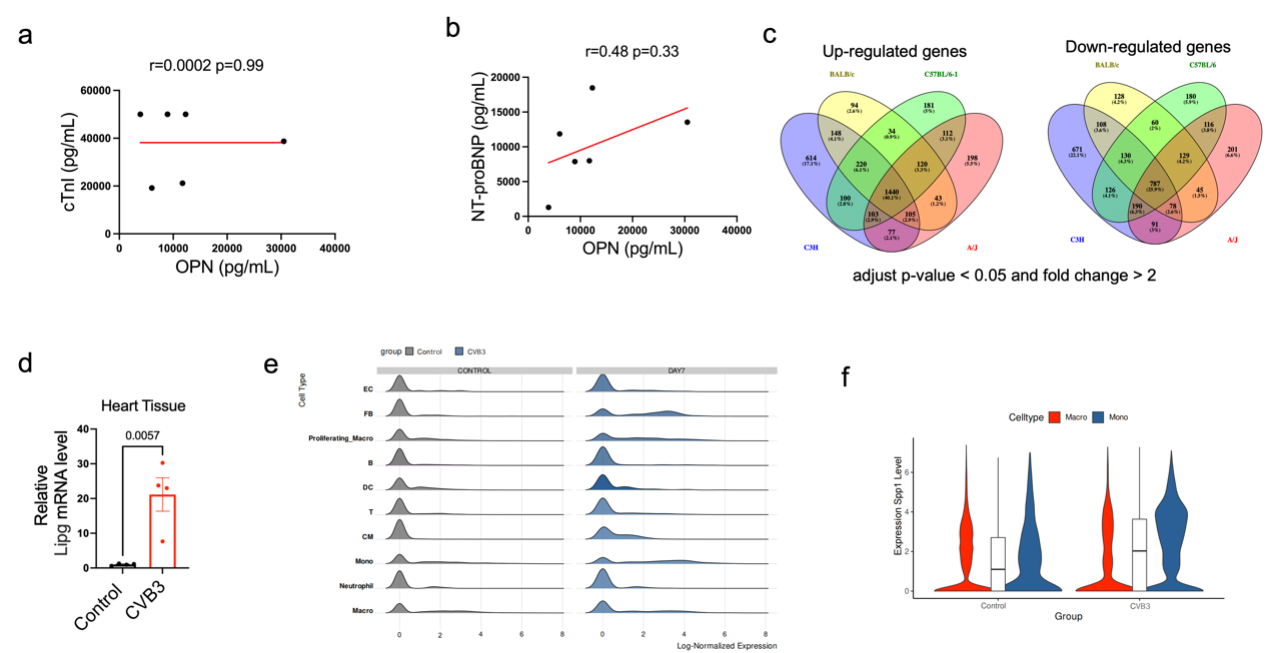
** **
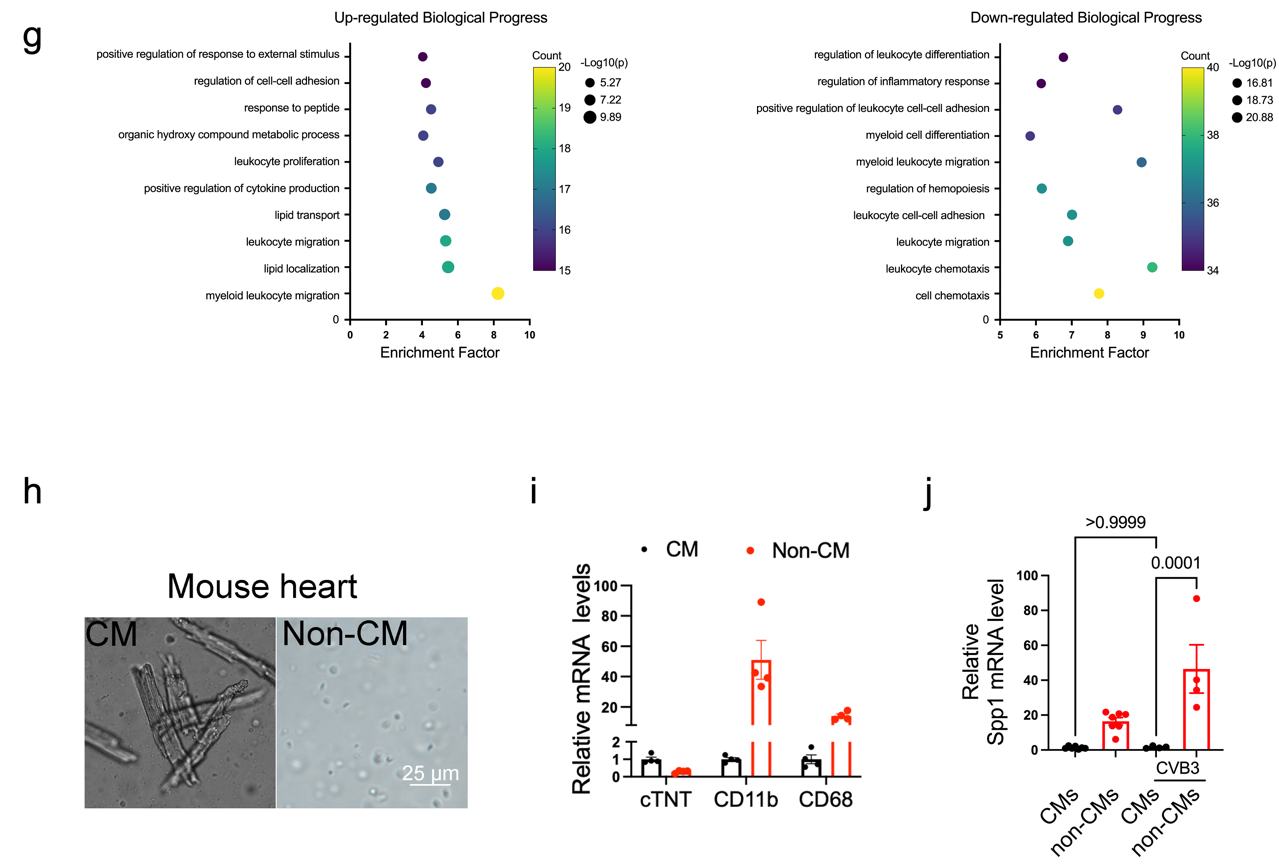
**

**Figure S1.** OPN expression in myocarditis.

a. Person analyses of the correlation between OPN levels and troponin I, respectively (N = 6).

b. Person analyses of the correlation between OPN levels and NT-proBNP, respectively (N = 6).

c. Veen diagram dysregulated genes among four myocarditis mice models.

d. qPCR analysis of Lipg expression in heart tissues from myocarditis mice models. p-value analyzed by Unpaired T-Test (N = 4).

e. Ridge plots showing the normalized expression of Spp1 across cell types in Control and CVB3 treated mice.

f. Violin plots showing the significant upregulation of Spp1 expression in monocytes and macrophages by CVB3 infection.

g. GO analysis between Spp1^+^ macrophage and Spp1^-^ macrophage.

h. Morphology of cardiomyocytes and non-cardiomyocytes isolated from heart tissues.

i. qPCR analysis of cTnT, CD11b and CD68 expression in CMs and non-CMs from heart tissues.

j. qPCR analysis of OPN expression in cardiomyocytes and non-cardiomyocytes from myocarditis mice models. p-value analyzed by two-way ANOVA with Tukey's multiple comparisons test (Control: N = 7; CVB3: N = 4).

**Supplementary Figure 2**


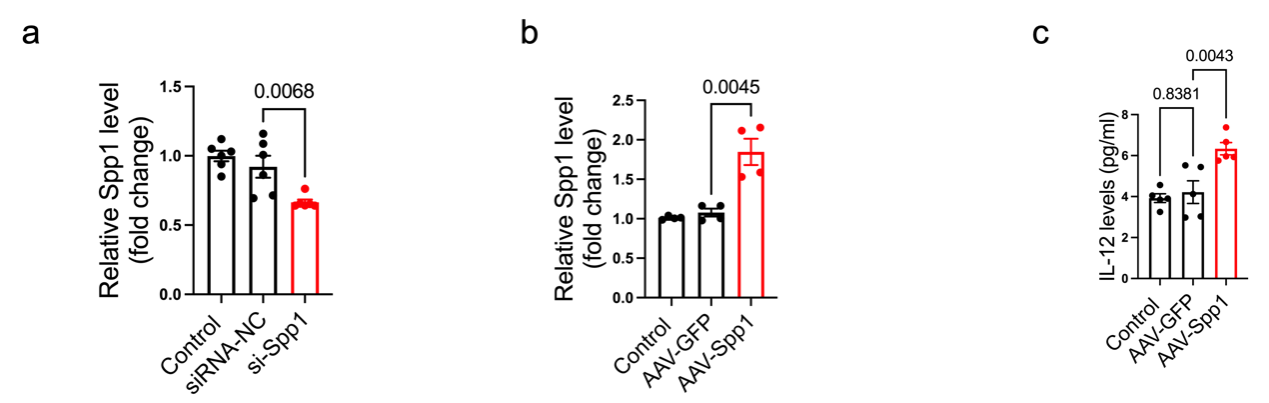


**Figure S2.** Spp1 expression in RAW264.7 macrophages.

a. qPCR analysis of Spp1 expression in macrophages after Spp1 knockdown. p-value analyzed by one-way ANOVA with Dunnett's multiple comparisons test (N = 6).

b. qPCR analysis of Spp1 expression in macrophages after Spp1 overexpression. p-value analyzed by one-way ANOVA with Dunnett's multiple comparisons test (N = 4).

c. ELISA was performed to detect IL-12 levels in macrophages after Spp1 overexpression. p-value analyzed by one-way ANOVA with Dunnett's multiple comparisons test (N = 5).

**Supplementary Figure 3**


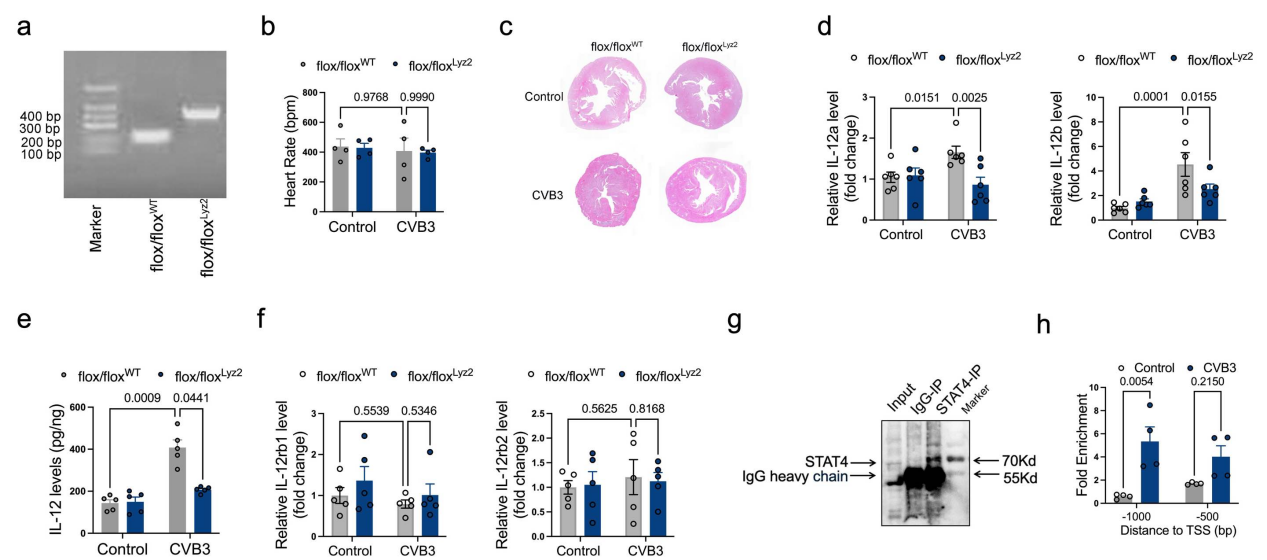


**Figure S3.** OPN function in acute myocarditis.

a. PCR assay confirm deletion of OPN in flox/flox^Lyz2^ mice.

b. Heart rate is unaffected in flox/flox^Lyz2^ or flox/flox^WT^ mice after CVB3 infection (N = 5).

c. H&E staining of inflammatory cell infiltration in heart tissue of flox/flox^Lyz2^ or flox/flox^WT^ mice after CVB3 infection.

d. qPCR analysis of IL-12a and IL-12b expression in flox/flox^Lyz2^ or flox/flox^WT^ mice after CVB3 infection. p-value analyzed by two-way ANOVA with Tukey's multiple comparisons test (N = 6).

e. ELISA assay for IL-12 expression in flox/flox^Lyz2^ or flox/flox^WT^ mice. p-value analyzed by two-way ANOVA with Tukey's multiple comparisons test (N = 5).

f. qPCR analysis of IL-12rb1 and IL-12rb2 expression in flox/flox^Lyz2^ or flox/flox^WT^ mice after CVB3 infection. p-value analyzed by two-way ANOVA with Tukey's multiple comparisons test (N = 6).

g. ChIP assay of STAT4 in macrophages (RAW264.7).

h. ChIP-qPCR assay of STAT4 binding on the promoter of Spp1. p-value analyzed by two-way ANOVA with Tukey's multiple comparisons test (N = 4).

**Supplementary Figure 4.**

**
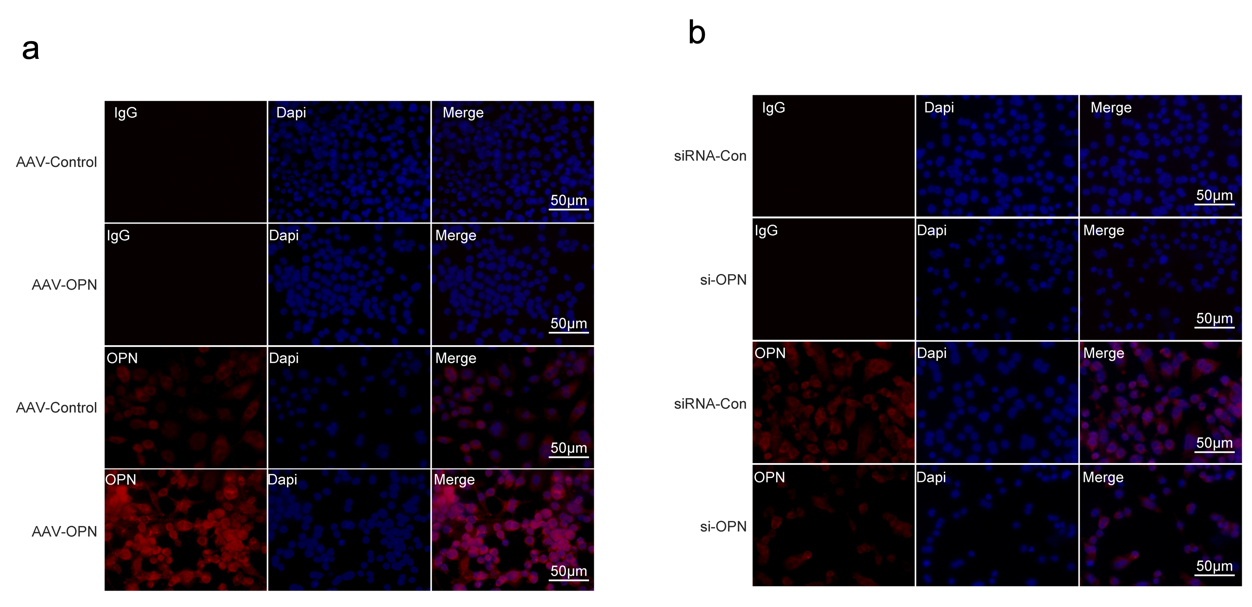
**

**Figure S4.** IF staining of OPN in RAW264.7 cells.

a. IF staining of OPN in macrophages with OPN overexpression.

b. IF staining of OPN in macrophages with OPN knockdown.

| **Supplementary Table 1.** The characteristics of patient with myocarditis and Controls. | | | | | | | | | |
| --- | --- | --- | --- | --- | --- | --- | --- | --- | --- |
| **Number** | **Sex** | **Age** | **HR**  **(bpm)** | **BP**  **(mmHg)** | **cTnI**  **(pg/ml)** | **BNP**  **(pg/ml)** | **OPN**  **(pg/ml)** | **EF**  **%** | **Diagnose** |
| Patient-1 | Male | 21 | 88 | 88/60 | 50000 | 8942 | 8942 | 26 | Myocarditis |
| Patient-2 | Male | 60 | 92 | 94/51 | 38729.4 | 30491 | 30491 | 36 | Myocarditis |
| Patient-3 | Female | 29 | 78 | 76/43 | 21186.5 | 11715 | 11715 | 23 | Myocarditis |
| Patient-4 | Female | 20 | 114 | 85/59 | 19159.8 | 6033 | 6033 | 41 | Myocarditis |
| Patient-5 | Female | 32 | 94 | 60/45 | 50000 | 12289 | 12289 | 31 | Myocarditis |
| Patient-6 | Male | 19 | 99 | 117/63 | 50000 | 3910 | 5232 | 28 | Myocarditis |
| Control-1 | Male | 31 | 76 | 120/78 | 0.4 | 89 | 4582.2 | 66 | Normal |
| Control-2 | Male | 55 | 82 | 130/82 | 1 | 125 | 4300.5 | 78 | Normal |
| Control-3 | Male | 42 | 67 | 102/82 | 0 | 87 | 7839.5 | 62 | Normal |
| Control-4 | Female | 22 | 80 | 119/90 | 0.8 | 142 | 345.3 | 72 | Normal |
| Control-5 | Female | 30 | 104 | 142/88 | 0.3 | 130 | 1986.6 | 68 | Normal |
| Control-6 | Female | 28 | 92 | 137/73 | 0.7 | 93 | 2422.5 | 79 | Normal |

**Note:** HR: heart rate; BP: blood pressure; cTnI: troponin; BNP: brain natriuretic peptide; OPN: osteopontin; EF: ejection fraction.

| **Supplementary Table 2.** The characteristics of patient with myocarditis. | | | | | | | | | |
| --- | --- | --- | --- | --- | --- | --- | --- | --- | --- |
| **Number** | **Sex** | **Age** | **HR**  **(bpm)** | **BP**  **(mmHg)** | **cTnI**  **(pg/ml)** | **BNP**  **(pg/ml)** | **OPN**  **(pg/ml)** | **EF**  **%** | **Diagnose** |
| Patient-1 | Male | 52 | 88 | 105/70 | 153 | 475 | 9023 | 45 | Myocarditis |
| Patient-2 | Male | 36 | 76 | 121/63 | 56 | 272 | 2983 | 18 | Myocarditis |
| Patient-3 | Male | 42 | 109 | 114/62 | 231 | 453 | 10234 | 40 | Myocarditis |
| Patient-4 | Male | 51 | 84 | 121/60 | 133 | 229 | 5982 | 46 | Myocarditis |
| Patient-5 | Female | 30 | 90 | 104/70 | 40 | 529 | 13084 | 45 | Myocarditis |
| Patient-6 | Male | 22 | 113 | 90/55 | 256 | 929 | 3910 | 48 | Myocarditis |

**Note:** HR: heart rate; BP: blood pressure; cTnI: troponin; BNP: brain natriuretic peptide; OPN: osteopontin; EF: ejection fraction.

| **Supplementary Table 3.** GSEA analysis for dysregulated genes after OPN overexpression. | | | | | |
| --- | --- | --- | --- | --- | --- |
| **Name** | **SIZE** | **ES** | **NES** | **NOM**  **p-value** | **FDR**  **q-value** |
| GOBP_NEGATIVE_REGULATION_OF_VIRAL_GENOME_REPLICATION | 53 | 0.71686 | 2.522543 | 0 | 0 |
| GOBP_NEGATIVE_REGULATION_OF_VIRAL_PROCESS | 73 | 0.6634294 | 2.4562027 | 0 | 0 |
| GOBP_RESPONSE_TO_TYPE_I_INTERFERON | 67 | 0.6583231 | 2.4241984 | 0 | 0 |
| GOBP_REGULATION_OF_VIRAL_GENOME_REPLICATION | 79 | 0.6304601 | 2.382676 | 0 | 0 |
| GOBP_INTERFERON_MEDIATED_SIGNALING_PATHWAY | 80 | 0.6043041 | 2.3098712 | 0 | 2.46E-04 |
| GOBP_REGULATION_OF_VIRAL_LIFE_CYCLE | 108 | 0.5640314 | 2.2499733 | 0 | 6.01E-04 |
| GOBP_ANTIVIRAL_INNATE_IMMUNE_RESPONSE | 59 | 0.6180888 | 2.2277381 | 0 | 8.57E-04 |
| GOBP_REGULATION_OF_VIRAL_PROCESS | 137 | 0.5181639 | 2.1392243 | 0 | 0.0035976 |
| GOBP_DEFENSE_RESPONSE_TO_VIRUS | 271 | 0.4726161 | 2.1233633 | 0 | 0.0038326 |
| GOBP_RESPONSE_TO_VIRUS | 338 | 0.4502867 | 2.1085923 | 0 | 0.0056691 |

| **Supplementary Table 4.** Binding sites of Stat4 on Spp1 promoter. | | | | | | | |
| --- | --- | --- | --- | --- | --- | --- | --- |
| **ID** | **Name** | **Score** | **Gene** | **Start** | **End** | **Strand** | **Sequence** |
| 1 | Stat4 | 14.651514 | Spp1 | 770 | 783 | + | attccaggaaataa |
| 2 | Stat4 | 11.989191 | Spp1 | 185 | 198 | - | gttcagggaaattc |
| 3 | Stat4 | 11.484244 | Spp1 | 271 | 284 | + | tttaaaagaaataa |
| 4 | Stat4 | 10.586589 | Spp1 | 263 | 276 | - | tttaaaagaaaaaa |
| 5 | Stat4 | 9.82818 | Spp1 | 767 | 780 | - | tttcctggaatata |
| 6 | Stat4 | 7.1336026 | Spp1 | 832 | 845 | + | attcagagaaccct |
| 7 | Stat4 | 6.8142824 | Spp1 | 613 | 626 | + | atttcgggaaaaga |
| 8 | Stat4 | 6.636564 | Spp1 | 614 | 627 | + | tttcgggaaaagaa |
| 9 | Stat4 | 5.346675 | Spp1 | 371 | 384 | + | cttcaaagtaataa |
| 10 | Stat4 | 5.308181 | Spp1 | 1760 | 1773 | + | cttccgggattcta |
| 11 | Stat4 | 5.003546 | Spp1 | 1562 | 1575 | + | ctgccgagacagca |
| 12 | Stat4 | 4.466965 | Spp1 | 141 | 154 | + | ctgcagtgaaaccc |
| 13 | Stat4 | 4.3269453 | Spp1 | 1979 | 1992 | + | attctgggaggtct |

| **Supplementary Table 5.** The primers for mice mRNA detection. | | |
| --- | --- | --- |
| **Name** | **Forward (5’-3’)** | **Reverse (5’-3’)** |
| OPN | GGTGGTGATCTAGTGGTGCC | ACTGCCAATCTCATGGTCGT |
| GAPDH | AGCTCATTTCCTGGTATGACAAT | GGCCCCTCCTGTTATTATGG |
| STAT4 | TGCAGAGAAGTCAACCACTTCA | TGGACTTGATTCCACTGAGACA |
| IL-12a | GAGATTCACCTCAGCTTCTCCTT | GTTTGGTCCCGTGTGATGTC |
| IL-12b | GAAGTCCAATGCAAAGGCGG | GAACACATGCCCACTTGCTG |
| IFN-γ | ACGGCACAGTCATTGAAAGC | TGTCACCATCCTTTTGCCAGT |
| IL12rb1 | TGTGTTTCTGAGCGTGGACA | CATGTCCATGAGGAGCCGAG |
| IL12rb2 | CGGGAAGAGCTCTGGAGAACC | GCTTTAATCAGCAGCCACATGAA |

| **Supplementary Table 6.** Primers for ChIP-PCR analysis. | | |
| --- | --- | --- |
| **Name** | **Forward (5’-3’)** | **Reverse (5’-3’)** |
| Primer-1 | GGCACTTGGGAGTTAGAATGAAA | ACACGAATTAGGGAGGTATAGGAATAG |
| Primer-2 | TATTAAAAGCAGGGTTTGGCAAG | CAGGCTTATTACCTTACATCTCCAAT |
| Primer-3 | GCAAGCATTCCAGTCTCACAAA | AGATAGAGCCCGCCTAAGCAA |
| Primer-4 | AGGGTCTGAAAGTTCTGCCGA | TGAAAGGTATGGATTCTCCTATCTAAGT |
| Primer-5 | TGACATCGTTCATCAGTAATGCTTT | ACCTGCTCCTACACTTCCTCCTC |
| Primer-6 | AGACTCGTGCTGTTCTGGTCTTTT | TTTTAACTTCTACATCCAACTGGCTT |
